# Group size, survival and surprisingly short lifespan in socially foraging bats

**DOI:** 10.1186/s12898-016-0056-1

**Published:** 2016-01-15

**Authors:** Yann Gager, Olivier Gimenez, M. Teague O’Mara, Dina K. N. Dechmann

**Affiliations:** Department of Migration and Immuno-Ecology, Max Planck Institute for Ornithology, 78315 Radolfzell, Germany; Department of Biology, University of Konstanz, 78464 Konstanz, Germany; International Max Planck Research School for Organismal Biology, University of Konstanz, 78464 Konstanz, Germany; CEFE UMR 5175, CNRS, Université de Montpellier, Université Paul-Valéry Montpellier, EPHE, 34293 Montpellier Cedex 5, France; Zukunftskolleg, University of Konstanz, 78464 Konstanz, Germany; Smithsonian Tropical Research Institute, Balboa, Ancón, Panama

**Keywords:** Cox proportional hazard model, Fitness, *Molossus molossus*, Multistate mark-recapture model, Social foraging, Sociality

## Abstract

**Background:**

The relationships between group size, survival, and longevity vary greatly among social species. Depending on demographic and ecological circumstances, there are both positive and negative effects of group size variation on individual survival and longevity. For socially foraging species in particular there may be an optimal group size that predicts maximum individual survival that is directly related to the potential for information transfer, social coordination, and costs of conspecific interference. Our aim was to investigate this central aspect of evolutionary ecology by focusing on a socially foraging bat, *Molossus molossus*. This species optimizes foraging success by eavesdropping on the echolocation calls of group members to locate ephemeral food patches. We expected to find the highest survival and longest lifespans in small groups as a consequence of a trade-off between benefits of information transfer on ephemeral resources and costs of conspecific interference.

**Results:**

In a mark-recapture study of 14 mixed-sex *M. molossus* social groups in Gamboa, Panama, spanning several years we found the expected relatively small and intermediate, but stable groups, with a mean size of 9.6 ± 6.7 adults and juveniles. We estimated survival proxies using Cox proportional hazard models and multistate-mark recapture models generated with recapture data as well as automated monitoring of roost entrances in a subset of the groups. Median survival of females was very short with 1.8 years and a maximum estimated longevity of 5.6 years. Contrary to our expectations, we found no relationship between variation in group size and survival, a result similar to few other studies.

**Conclusions:**

Strong selection towards small group size may result from psychoacoustic and cognitive constraints related to acoustic interference in social foraging and the complexity of coordinated flight. The short lifespans were unexpected and may result from life at the energetic edge due to a highly specialized diet. The absence of a relationship between group size and survival may reflect a similar but optimized survival within the selected range of group sizes. We expect the pattern of small group sizes will be consistent in future research on species dependent on social information transfer about ephemeral resources.

**Electronic supplementary material:**

The online version of this article (doi:10.1186/s12898-016-0056-1) contains supplementary material, which is available to authorized users.

## Background

Group living is widespread across the animal kingdom and evolved convergently from an ancestral solitary state in different taxa (e.g. [1]). Many species remain solitary or are only seasonally social [2], showing that sociality is only beneficial when benefits outweigh the costs [3]. For example, in the social cliff swallow (*Hirundo pyrrhonota*), colony size is correlated with at least 13 different types of costs (e.g., parasitic infestation, brood parasitism) and at least 13 different types of benefits (e.g., predator-avoidance, information transfer, [4]). Thus, group size is an important trait that responds to cost-benefit regimes depending on a species, its ecological niche and life history [4, 5]. In fact, the size of animal aggregations can vary from small social groups below ten individuals like the prides of lions [6] to huge colonies with millions of seabirds or bats [7, 8]. Thus, a crucial step in any study is to distinguish between aggregations of individuals, due to external circumstances such as roost limitation, and “true” social groups with reciprocal relationships (which may be contained in larger aggregations) [9].

Sociality should be adaptive [3], we therefore expect fitness benefits of optimal group size resulting in prolonged survival, enhanced reproductive success or both. Life history theory predicts that animals should allocate their energy differently to individual reproduction or survival [10, 11]. As a general rule of thumb, small animals are short-lived and produce many offspring (e.g. rodents, *r*-strategists) while large animals are long-lived and have few offspring (e.g. elephants, *K*-strategists) [12]. Bats are an exception to this general pattern, being small but long-lived and producing relatively few offspring. However, while life history theory does not incorporate sociality, there are many studies linking group size with survival. Different parameters are used to investigate this, the two most common being maximum lifespan (or maximum longevity) and an averaged estimate for the survival of the group members. Comparative studies on birds and mammals did not find any correlation between maximum lifespan and group size [13–16]. The same is not true for the relationship between group size and survival. Group size is often positively correlated with survival in many taxa, including termites [17], social spiders [18], birds [19–21] and mammals [22, 23]. In all of these examples, social behaviours, such as predator avoidance, social thermoregulation or social foraging, lead to improved survival. However, there is a limit to the benefits of increasing group size. For instance in certain colonies of Neotropical spiders, survival of the colonies increased with colony size. But above a threshold in colony size (~15 individuals), survival of the colony decreased, presumably because of an increase in intra-colony competition [18]. In other species, such as the Seychelles warbler (adults) and a social spider (juveniles), there is even a strictly negative relationship between survival and group size, again probably due to competition for resources [18, 24]. Despite this decreased survival, increasing group size brings reproductive benefits in the Seychelles warbler. The reverse situation was observed in Neotropical spider, with survival benefits but reproductive costs with increasing group size leading to a trade-off situation and resulting in maximum fitness at intermediate size [25]. Finally, in some species, including wild dogs, juvenile rodents, primates or coatis, group size and survival are independent [26–29], interpreted to be a result of specific ecological conditions such as low competitor density and high food availability.

One important benefit of sociality is information transfer between individuals [30–32]. In a foraging context, animals can detect conspecifics present at food patches through “local enhancement” [33]. The number of animals at a food patch and the modality of the information they use (e.g., sound, vision, olfaction) can have crucial implications for their fitness. Many bird species rely on local enhancement through vision to detect conspecifics at a food patch (e.g. seabirds, vultures, ospreys and swallows) [34–37]. In an empirical test of recruitment of seabirds to food patches, adjusted estimates for average distance recruitment ranged from 4.9 to 11.3 km [35]. Therefore, vision, the most commonly used mode of information transfer during foraging leads to the attraction of individuals over long distances and is believed to have led to the evolution of bird colonies [38]. Echolocating bats, in contrast, cannot use vision during nocturnal foraging. Instead they benefit from information transfer by eavesdropping on changes in each others’ echolocation calls that indicate successful localization of a food source [39–44]. Compared to vision, the propagation distance of echolocation calls is very short due to rapid atmospheric attenuation [45]. For instance, maximum hearing distance of conspecifics was estimated at 54 m in *M. molossus* and 35–40 m in *Noctilio albiventris* [40, 41], however this is ten times the distance from which they can actively localize a prey item. The restriction to different modalities (e.g. vision vs. sound) therefore has direct implications for the foraging strategy. However, the relationship between social foraging, the composition of groups, survival, and group size remains poorly understood in bats despite the wide reliance on social information to locate resources and its effect on the evolution of group living in bats and other animals.

To test if and how social group size affects survival in bats, we studied Pallas’s mastiff bat *Molossus molossus* (Pallas, 1766), a species that forms stable social groups that roost and forage together [41]. The narrow-shaped wing morphology of *M. molossus* results in high energetic requirements within an open-air foraging niche [46]. As a result of this specialized morphology, this species depends on ephemeral insect swarms as their only food source that are only available at dawn and dusk, and therefore foraging time is restricted to short activity peaks of less than an hour [41]. These energetic and morphological limitations as well as the narrow foraging niche make increased foraging efficiency through the use of social information from conspecifics highly important. One might thus expect that a large number of foraging partners and correlated increase in social group size would be advantageous. However, theoretical work indicates optimal individual uptake in groups with a small number of signallers in a recruitment scenario [47]. Due to the short availability window of its resource and the modality of information transfer (acoustical), *M. molossus* must coordinate flight and eavesdrop on echolocating group members on the wing instead of recruiting. This quickly creates a complex system of signallers and receivers and thus a trade-off between benefits of improved indirect prey detection and costs of conspecific acoustic interference [48]. Thus, we hypothesized that there is an ideal group size for *M. molossus,* and that this group size should be small. Individual survival should then be highest in these ideal small groups. To test this we used two approaches: (a) We captured 14 social groups multiple times over several years. During a subset of this time period, (b) we also monitored four of these groups with automatic transponder readers to get a more precise temporal resolution of changes in group composition. We modelled the role of group size using these two data sets in two survival analyses based on the Cox proportional hazard and multistate mark-recapture models. We based our survival analysis on all adults present in a group because males and females are known to forage together [41]. However, we only analysed survival and lifespan for adult females because males probably spend time as bachelors before and possibly even after their presence in the groups, which then is probably only indicative of their harem tenure. The unique access to data from free-ranging socially foraging bats thus allowed us to test predictions from theoretical evolutionary models in a system of naturally behaving animals.

## Results

### Life cycle of *Molossus molossus*

#### Captures

We collected data during 81 capture events of 14 social groups and a total of 490 individuals (adults and juveniles). We recaptured only a subset of the bats we marked as post-dispersal adults (121 females and 31 males) and none of them were observed switching roosts over the entire study period (maximum of 4.3 years). We caught pregnant females between March and August and observed a pregnancy peak in April. Anecdotal data indicate a second minor birth peak at the end of the year, but we cannot confirm this. Based on capture data, we assume that females disperse from the social group to settle permanently in a social group. It also appears that they can start reproducing directly after natal dispersal. We cannot infer the complete life cycle of males based on the capture data but we suspect a bachelor phase between natal dispersal and tenure of harems as well as after the end of tenure [49, 50].

#### BaTLis

**Fig. 1:**
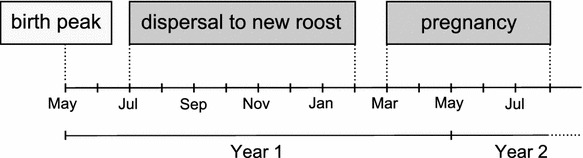
Life cycle of female *Molossus molossus*

Data from the BaTLis (automated transponder readers custom-made by the workshop of the University of Konstanz) are consistent with captures. In addition, activity of unmarked individuals on the BaTLis suddenly increased around June, and this activity peak corresponded with juvenile fledging. We monitored a subset of 24 juvenile females and 19 juvenile males with the BaTLis. They were marked between July and September and were last detected by the transponder readers between July of the same year and February of the following year indicating their death or natal dispersal. The life cycle of female *Molossus molossus* is illustrated in Fig. 1.

### Variation of group size

#### Captures

**Fig. 2:**
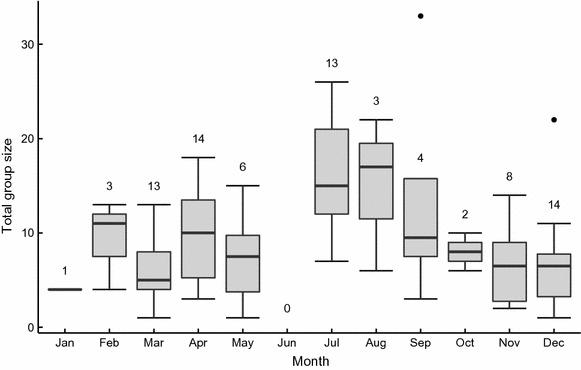
Temporal variation of total group size of *M. molossus* (including juveniles). *Box plots* represent from bottom to top: minimum, lower quartile, median, upper quartile and maximum. *Dots* indicate observations further than one SD away from the mean. The numbers of social groups caught per month are indicated above the *boxplots*

We found that total size of groups we caught (adults and juveniles) was small compared to the know range of bat colonies, ranging from one to 32 individuals, with a median of eight and a mean of 9.6 ± 6.7 individuals. Single individuals were caught only on three occasions: the same adult male on two occasions (roost 164) and an adult female on one occasion (roost 152A). We suspect other individuals were inside the roost but did not emerge because we captured several individuals during other capture events of these groups. Adult group size varied between one and 25 individuals, with a median of seven and a mean of 8.1 ± 5.1 individuals. Adult sex-ratio was biased towards females with a median of 78 % and mean of 71 ± 26 %. The number of adult males was one or two in 75 % of the capture events. Group size increased during July and August (Fig. 2), coincident with juvenile fledging.

#### BaTLis

Adult group size varied between three and 13 individuals. We found very similar mean and SD for group size: Group 1: 9.0 ± 2.3, Group 2: 10.0 ± 2.5, Group 3: 10.9 ± 3.9 and Group 4: 11.1 ± 2.6 individuals.

### Estimates of female lifespan

We followed the 14 social groups of the study between 0.5 and 4.7 years. We recaptured 30.2 % of the 280 females marked as adults (n = 124, one to seven recaptures per individual). Lifespan of adult females from marking time ranged from five to 1709 days (4.7 years) with a median of 280 days (0.8 years). Corrected estimates from the closest birth peak expanded lifespan between 132 and 2044 days (0.4–5.6 years) with a median of 646 days (1.8 years).

### Predictors of female lifespan and monthly survival

#### Cox proportional hazard model using capture data

We filtered out 44 % of the captures when including only adult females that had immigrated since last capture. This resulted in 70 adult females with 114 recapture events. Corrected lifespan estimates (from potential birth to last capture) ranged here from 132 days (0.4 years) to 1210 days (3.3 years) with a median lifespan of 436 (1.2 years). The selected variables (adult or total group size) complied with the test of proportionality and had no statistical influence on survival estimates for both survival datasets (Table 1). 24 females survived less than a year (34.3 %), 30 between 1 and 2 years (42.9 %), 8 between 2 and 3 years (11.4 %) and 8 other between 3 and 4 years (11.4 %).

**Table 1:** Results from the two Cox proportional hazard survival models

Survival dataset	Variable	Significance	Proportionality
Assumed birth to last capture	Total group size	0.37	0.08
Assumed birth to last capture	Adult group size	0.11	0.58

The significance of the predictor variable and test of proportionality are based on scaled Schoenfeld residuals

#### Multistate mark recapture models using BaTLi data

Four groups were randomly selected based on whether it was possible to install a BaTLi at the entrance. We used the transponder reader data to investigate the influence of a set of variables on adult female survival (n = 63). As we monitored these groups only for 15 months, the estimates here represent monthly survival rather than complete lifespans. Size of the four focal groups changed over time, ranging from three to 13 adult males and females but with very similar mean and SD for group size. We divided the range of group sizes into three categories to obtain higher confidence in our monthly survival estimates (“small”: 3–6, “medium”: 7–9, “large”: 10–13). Multistate models were found to adequately fit the data for the four groups (Group 1: χ^2^ = 4.348, df = 3, P = 0.226; Group 2: χ^2^ = 5.823, df = 8, P = 0.667; Group 3: χ^2^ = 3.083, df = 2, P = 0.214; Group 4: χ^2^ = 1.490, df = 1, P = 0.222). The best fitting models, ordered by lower ΔQAICc, were (1) adult group size, (2) marking year, (3) the null model, (4) group ID (1–4), and (5) the month of first capture (Table 2). The best fitting model was strongly supported, with a ΔQAICc of 118 units in comparison to the next-best model (marking year). Note that while performing goodness-of-fit tests, we could not test for trap-dependence because of a lack of data. We reran the same set of models modified to incorporate a trap-dependence effect. Specifically, we had a detection probability function of an individual covariate “captured” or “not captured” at the previous event. The ranking of the models and survival estimates from the best-fitting model here were similar to the analysis not incorporating trap-dependence on the detection probability, therefore we show only the results from the model without trap-dependence.

**Table 2:** Multistate mark-recapture models of survival for *M. molossus*

Model	QAICc	ΔQAICc	Number of parameters	Deviance
(1) IS[gs]. Ф[gs]. ѱ[gs]. P[.]	1380.0	0.0	12	1407.5
(2) IS[gs]. Ф[marking year]. ѱ[.]. P[.]	1498.0	118.0	9	1480.0
(3) IS[gs]. Ф[.]. ѱ[.]. P[.]	1509.4	129.4	5	1499.3
(4) IS[gs]. Ф[social group]. ѱ[.]. P[.]	1513.5	133.5	8	1497.2
(5) IS[gs]. Ф[month]. ѱ[.]. P[.]	1522.9	142.9	18	1485.6

The survival estimates are based on 63 adult females from four social groups. The five models are ordered by the ΔQAICc where a lower value indicates a better fit of the model to the data. These models estimated initial state (IS), survival (Ф), transition probabilities (ѱ) and constant detection probability (P) for the predictor variables adult group size (gs), marking year, social group and observation month

**Fig. 3:**
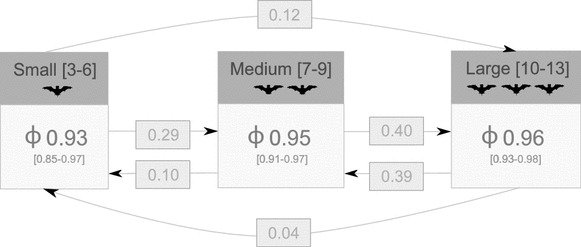
Multistate mark-recapture model for survival and adult group size in *Molossus molossus*. Survival estimates Ф for group size categories (small, medium and large) and transition probabilities ѱ between these categories are depicted. For example, the survival probability (from month t to t + 1) in a small group is 0.93 (95 % confidence interval 0.85–0.97), and the probability that a small group will transition to a large group (from month t to t + 1) is 0.12. These parameters were estimated with the multistate mark recapture model with group size including initial state of group size IS[gs] and detection probability P[.] in the model IS[gs]. Ф[gs]. ѱ[gs]. P[.]

Based on the best-fitting model (adult group size), we estimated survival for the three categories of group size. We found similar probabilities of monthly across categories (Fig. 3): “small” groups (3–6 bats; 0.93, 95 % confidence interval (CI) 0.85–0.97), “medium” groups (7–9 bats; 0.95, 95 % CI 0.91–0.97) and “large” groups (10–13 bats; 0.96, 95 % CI 0.93–0.98). The confidence in survival estimate is lower for “small” groups because we had only 10 group-month observations for this category, while we had 24 and 26 for “medium” and “large” groups, respectively. With the same model, we obtained a detection (or recapture) probability of P[.] of 0.95. We also obtained transition probabilities between group size ѱ[gs]—the probability that the group size an individual occupies changes from 1 month to the other—ranging from 0.04 to 0.40 (Fig. 3). The most frequent group size transitions occurred between the two most frequent group sizes (“medium” and “large”), with transition probabilities per month of 0.40 (medium to large) and 0.39 (large to medium). There was no influence of temporal variation of group size on monthly survival probability.

### Estimates of tenure length for harem males

We recaptured 22.5 % of the 107 males marked as adults (n = 31, one to four recaptures per individual). Time from marking to last recapture ranged from six to 1076 days (2.9 years) with a median of 228 days (0.6 years).

## Discussion

Our data suggest that even though aggregations of bats can consist of up to several million individuals, there is strong selection for small group size (≤25 adults) in *Molossus molossus*. There was little variation of group size and short median female survival of 1.8 years (corrected estimates from all recaptured data). Furthermore, group size was not correlated with longevity as calculated from recaptures and monthly survival as calculated from automated monitoring in a subset of groups (i.e. BaTLis).

Thus, along with our predictions, *M. molossus* forms small groups, composed of a stable core of adult females and one adult male in most groups (mean of 8.1 ± 5.1 adults). BaTLi data showed that all juveniles of both sexes dispersed from the natal group. Once they settled in a new group, adult females remained there longer than adult males (median of 0.8 vs. 0.6 years, maximum of 4.7 vs. 2.9 years). Thus, the cluster of adult females emerges as the stable and primary unit of *M. molossus* social organization. Stable clusters of females favour the evolution of a male strategy of female-defence polygamy [51, 52]. Where females live in social groups, one single male is commonly responsible for most or all mating, as shown for instance in primates, antelopes or bats [49, 53–55]. Our data suggest that maximum male tenure length (2.9 years) exceeds the sexual maturity of the sired daughters, driving natal dispersal of juvenile females to avoid inbreeding with their father [56]. This phenomenon is observed in few other species of mammals [57] but seemingly not uncommon in tropical bats living in stable groups [58, 59]. Natal dispersal of juvenile males may result from an eviction by the harem male (potentially their father) and/or an attempt to find mating opportunities [60, 61]. We are unable to estimate lifespan for male *M. molossus* because we do not know the time from natal dispersal to harem take-over as well as the fate of replaced harem males. We suppose males remain solitary or join a bachelor group before and after harem tenure as already observed in other tropical bats [49, 50] but the roost preferences of these males seem to differ from those of females as we did not find them during our exhaustive roost surveys in buildings. A challenge for future studies will be to follow males throughout their life cycle to obtain realistic lifespan estimates. Similar to other polygynous mammals, we expect to find reduced longevity in adult males resulting from intense male–male competition and weaker selection for longevity [62].

Group fidelity of adult females was very strong as shown by one to seven recaptures of 121 females in the roosts where they were initially marked as adults. In the roost we monitored the longest time, a female was recaptured after 4.7 years with an estimated lifespan of 5.6 years. We expect that maximum lifespan records might still increase slightly with a longer study period, but we are confident that our mean estimates of 1.2 and 1.8 years are representative for the species. This is unexpected, because bats are famous for exceptionally long lifespans relative to their small body mass (i.e. allometry of lifespan), the record being held by a 8-g insectivorous *Myotis brandtii* which was recaptured after 41 years in the wild. On average, lifespan of bats is around 3.5 times longer than in non-flying mammals after correcting for body mass [15]. Maximum lifespan for two other molossid species with a similar ecological niche as *M. molossus* is 12 and 13 years, respectively for the much larger European *Tadarida teniotis* (mean mass of 30 g) and the American *T. brasiliensis* (mean mass of 12.5 g similar to our study species) [63–65]. The higher longevity observed in these two species may result from decreased predation associated with cave roosting [15] and/or a broader foraging niche in terms of prey as well as temporal and spatial food availability. Despite its relevance for population dynamics, maximum lifespan represents only the upper limit of the survival curve. Better knowledge about average or median lifespans appears to be important, but often lacking life-history parameter in bat studies (but see [66]) for the better understanding of the pace and shape of the survival curve [67, 68]. Our data revealed a skewed survival curve for females, with median survival of 1.8 years and a maximum longevity of 5.6 years. Low values for median and maximum lifespan in *M. molossus* may result from life at the energetic edge due to a narrow foraging niche. This bat is an open-air forager, with long and narrow wings that result in high wing loading and high energetic costs of flight [69, 70]. This bat is also specialized to forage on insect swarms which are abundant when found but remain relatively unpredictable in space and time [39]. The species shows a bimodal activity pattern, after sunset and before sunrise [71, 72]. The predominant foraging activity occurs after sunset, sometimes for only a half an hour interval [71, 72]. This limited burst of activity probably result from a peak in insect density [70]. Because insect patches can be dispersed by wind and rain, bats sometimes entirely skip a night of foraging. To limit the risk of starvation, this species can maximize energy intake by socially foraging [41] and minimize energy investment by lowering metabolism when roosting [73]. However, due to the short foraging window, the unpredictability of the resource and the flight costs, these bats have a risk of starvation. Our data, suggesting that most females *M. molossus* only reproduce once or twice in their short lifetimes, is consistent with this hypothesis. Further investigation will be necessary to determine how the small percentage of longer-lived individuals contribute to the maintenance of the species, what causes these enormous variations in lifespans and how they are linked to the ecology of species. In addition, it will be important to find other factors influencing variation in female lifespan, such as foraging efficiency, and also following up on anecdotal reports about twinning as well as an additional smaller reproductive peak to better understand how stable populations of this species can persist.

Social groups of *M. molossus* are stable year-round, implying that benefits of sociality permanently outweigh the costs [3]. Foraging benefits via information transfer about ephemeral resources have been postulated as a major reason for coloniality in seabirds [74, 75]. And similarly, in *M. molossus* and other bats, the main benefit of group living is probably increased foraging efficiency through acoustic information transfer about ephemeral insect patches [39–41]. The daily availability of *M. molossus*’ food source is so short that information must be shared in the foraging arena on the wing, most likely via acoustic eavesdropping [40]. This means that groups must coordinate flight and filter relevant information from the echolocation calls of their social partners. Even though we do not understand yet how this works in detail, such a network of signallers and receivers quickly becomes very complex and confusing. Our results confirm our hypothesis that this should exert strong selection pressure on small group size because the 14 groups were ranging between one and 25 adults. This is in contrast to opportunistically eavesdropping species that do not emerge in coordinated flight and maintain group cohesion throughout their foraging period [76, 77].

While our expectations regarding small group size were confirmed, we did not find that individual lifespan was influenced by variation of group size. In both datasets (capture and BaTLi data), group size had no effect on lifespan or monthly survival despite group size being the most explanatory variable in the multistate mark recapture analysis. We postulate that selection on group size is so strong that the resulting variation, mainly caused by the brief appearance of pre-dispersing juveniles, is too small to have an effect on the adult females in the group. In fact, a closer look at previous work that found no or a negative relationship between group size and survival reveals consistently small group sizes in animals with complex social systems (≤33 individuals), e.g. 1–6 individuals in the Seychelles warbler [24], 2–12 adults in degus [29], 2–10 adult females in the coati [26], 2–17 individuals in the wild dog [28] and 6–33 individuals in the leaf monkey [27]. This suggests that selection for small group size in complex social systems may be fairly common. In other species, the limited variation of group size observed here (3–13 individuals) can still cause survival differences that can be detected. For example in the Seychelles’ warbler, survival is negatively correlated with group size (one to six individuals) [24].

The apparent absence of relationship between group size and survival in *M. molossus* could have technical and/or biological explanations. Our estimates of monthly survival for the three categories of group size were associated with narrow confidence intervals (respectively 0.85–0.97, 0.91–0.97 and 0.93–0.98). A dataset including more groups and individuals may lower confidence intervals and reveal survival differences that remained hidden so far. Alternatively, our results correspond to the reality and selection on ideal group size is so strong that the remaining low variation does not affect survival. In the framework of life history, the relationship between group size and reproduction remains to be investigated in future studies.

Bats are well known for roosting in large or even gigantic colonies, e.g. the closely related molossid species *Tadarida brasiliensis* that occurs in caves numbering up to tens of millions of individuals [7]. A confounding effect here may stem from the fact that many bat species are highly dependent on suitable, but limited roosts. *Molossus molossus*, too, may roost in such larger aggregations (300 individuals or more, [78]), composed of several social groups at other study sites where roosts are more limited. Several bat species, such as *Myotis bechsteinii* [79], *Nyctalus lasiopterus* [80] or *Eptesicus fuscus* [81], form fission–fusion societies. This allows animals to have access to the knowledge of a large pool of individuals but the daily subgroups are relatively small as a flexible reaction to social and environmental conditions. This may also mediate other detrimental effects of group living in bats such as parasite or disease transmission rates or competition [82, 83]. Similar societies are found in a great diversity of taxa, e.g. house sparrows [84], chimpanzees and leaf monkeys [85], or spotted hyenas [86]. However, most socially complex bat species form smaller and more stable social groups similar to *M. molossus* (≤25 adults), e.g. the molossid *Tadarida pumila* [87], other socially foraging bats [40, 49], tent-making bats [88–90] and other roost-making bats [50, 91]. We expect that with future research this will be a consistent pattern, especially in species that are ecologically dependent on information transfer about the location of ephemeral resources.

## Conclusions

In summary, this study suggests strong selection for small groups (≤25 adults) in a socially foraging bat. Our results are in agreement with models of recruitment on ephemeral resources suggesting a small and stable range of signalers in the groups optimizes individual uptake [47]. In our in situ eavesdropping scenario, where every individual is a signaler and receiver at a same time, the same selection pressure seems to apply to optimize trade-off between foraging benefits from information transfer and acoustic confusion impairing prey detection performance [48]. Our alternative survival analyses based on free-ranging animals independently found no effect of group size on survival, a pattern found in few similar studies and potentially resulting from life at the energetic edge due to a highly specialized diet. We expect similar results for future research conducted on species dependent on information transfer and ephemeral resources.

## Methods

### Data collection

We collected data in Gamboa, Panama (09°07′N 79°41′W), where *Molossus molossus* roosts in crevices in houses. We defined social groups as the set of individuals roosting in a single building crevice, but sometimes several social groups occupied separate crevices in the same building. We collected data about group size in two ways: repeated captures from roosts and automated monitoring with transponder readers (henceforth called “BaTLis”, custom-made by the workshop of the University of Konstanz).

#### Captures

We captured social groups with mist nets (Ecotone, Gydnia, Poland) at the entrance of roosts during evening emergence. The nets formed a closed space around the roost entrance, thus the entire group was caught in most cases apart from individuals that potentially remained in the roost. Between 2008 and 2014, we caught 14 mixed-sex social groups, resulting in 81 capture events and 490 individuals (2–9 capture events per group). We determined sex, age and reproductive status, and individually marked all bats with a subcutaneous passive integrated transponder (Trovan ID-100, Euro ID, Weilerswist, Germany) at first capture.

#### BaTLis

We monitored the roost entrance of four of the 14 groups with the BaTLis between April 2013 and June 2014 and followed the presence of each marked bat. Each BaTLi contained two light beams to determine direction of individuals passing as well as a balance. These two latter datasets allowed us to follow and estimate the number of unmarked bats using the roost entrance, e.g., immigrants into the group as well as freshly fledged juveniles. Over the 15 recorded months, these four roosts were recaptured 4–5 times each to mark new individuals with a maximum interval of 6 months between recaptures. Capture and handling of animals were carried out with permission from the Autoridad Nacional del Ambiente in Panama with approval from the Institutional Animal Care and Use Committee of the Smithsonian Tropical Research Institute (2012-0505-2015, [92]). The species investigated, *M. molossus*, was not considered endangered.

### Life cycle of *Molossus molossus*

#### Captures

We first determined the proportion of adults recaptured and checked if they switched roosts. We identified the periods of pregnancy and appearance of fledged juveniles using the capture data.

#### BaTLis

With the BaTLis, we were able to additionally monitor the timing of the increase of untranspondered individuals passing the entrance, indicative of a cohort of freshly fledged offspring. This allowed us to evaluate the timing of birth. Juveniles we caught and marked after fledging were used to determine the timing of juvenile dispersal as determined by the BaTLis.

### Variation of group size

#### Captures

We calculated total group size (adults and juveniles) and adult group size at each capture to assess the temporal variation of group size (see Additional file 1 for raw data). We also determined the proportion of adult males and adult females.

#### BaTLis

The BaTLis allowed a higher temporal resolution of changes in group size caused by death, immigration, or harem male replacement, but only over the shorter time period of 15 months when BaTLis were employed.

### Estimates of female lifespan

Our capture data showed that *M. molossus* lives in harems with regular replacement of the harem male, but stable female social groups (see “Results”). Based on the switch from pregnant to lactating females as well as calculating back roughly 1 month from the time we first caught freshly fledged offspring, we determined that the major birth peak occurs in May. From the disappearance of marked female subadults from groups, we were able to tell that all offspring disperse within 1–8 months of fledging. We then estimated lifespan by filtering the capture data in the following way: for each unmarked bat we captured, we determined if there had been a previous capture of the same roost where this individual had not been present. This meant that it had dispersed from its natal group and immigrated since the last capture. Thus, we started counting its lifespan from the previous May as a conservative minimum estimate. For example, a female marked in November was considered born in May of the same year, resulting in a lifespan correction of 7 months. We found no adult females that changed group, therefore we assumed that adult females’ disappearance from a group indicated their death.

### Predictors of female lifespan and monthly survival

We used two different survival analyses, a first analysis based on the capture dataset and a Cox proportional hazard survival model (Cox PH) and a second analysis based on the BaTLi dataset and multi-state mark recapture survival models (MSMR).

#### Cox proportional hazard model using capture data

These survival analyses are less robust because they do not take into account detection probability and changes in group size but we included more females over a longer time period in this dataset (n = 70 over a maximum of 2.5 years after filtering) and we could estimate lifespan. We used the estimates derived from the capture data: time between estimated birth and last capture as described above, i.e. lifespan (see Additional file 2 for raw data). Survival data were right-censored when the female was still alive at the last capture event. We used the Cox PH model [93], based on continuous time and the assumption of perfect detection (100 % probability of capture) and available in the R package *survival* [94]. We built two models combining the lifespan estimates and two predictor variables: total group size and adult group size. In these models, the individual survival probability at the recapture event [t] was based on the group size (total or adult) of the individual’s group during the previous recapture event [t − 1]. We tested the two models for the proportional hazard assumption of the predictor variables based on the scaled Schoenfeld residuals also using the R package *survival*. We also split the survival range into yearly categories (0–1, 1–2, 2–3 and 3–4) and determined the number and proportion of females for each of them.

#### Multistate mark recapture models using BaTLi data

In this second analysis, fewer females were analysed over a shorter time period (n = 63 over 15 months) but the models implemented detection probability and transitions in group size. We investigated predictors of monthly female survival using BaTLi data from the four groups (n = 63 adult females, see Additional file 3) and multistate mark recapture models (MSMR, [95]). These models are Markovian (conditional on the present state of the system, its past and future are independent) and rely on discrete time categories (e.g. calendar month) that we used to model temporal change in individual state (e.g. group size). With the MSMR models, we simultaneously estimated initial state (i.e. group size whenever first captured), survival probability, changes in group size, as well as detection probability for one or several predictor variables (e.g. social group, observation month). Detection probability (P) is a crucial parameter, often smaller than one and highly variable in natural populations, which can lead to flawed biological conclusions when not considered in mark-recapture analyses [96].

Our dataset consisted of 63 rows (one for each adult female) and 15 columns (one for each month of the study period). Each cell of the matrix contained either a “0” when the focal individual was absent or recorded the adult group size, when the focal individual was present. We categorized group size into “small” (3–6 individuals), “medium” (7–9) and “large” (10–13) to obtain higher confidence in the survival estimates. We assigned an age (i.e. marking year) and social group to each female. Although we observed occasional brief visits of adult females into neighbouring roosts (n = 9 events), no adult female was ever observed to permanently change groups and we therefore assumed stable group identity.

We performed multisite goodness-of-fit tests on the adult females dataset [97] using the software U-Care v. 2.3.2. [98]. We modelled monthly survival based on this survival matrix and five MSMR models. Each model comprised the following parameters (see also Table 2): IS[gs] or initial state for adult group size (the percentage of individuals initially observed in “small”, “medium” and “large” groups, the same in all models), Ф or survival probability (i.e. from one month to the next), ѱ[.] or group size transition probability (implemented in one of the five models, see below) and a constant detection probability P[.]. Preliminary investigation showed that the use of more than one predictor variable caused high uncertainty in the estimates. Consequently, we only estimated survival (Ф) using a single predictor variable per model: (1) adult group size (gs), (2) social group identity, (3) month of the study, (4) marking year, and (5) a null model without predictor variable. The first model, estimating the effect of group size, also incorporated a transition probability between group sizes ѱ[gs]. In this model, an individual survival probability at month [t] was based on the size of the individual’s group during the previous month [t − 1]. Model selection was performed using the program E-SURGE [99] with the Akaike Information Criterion corrected for small samples (AICc) as a measure of the trade-off between goodness of fit and complexity of the model. A threshold of 10 AIC units of difference was used to select the best-fitting model [100].

### Estimates of tenure length for harem males

We estimated potential tenure length of harem males by calculating the time interval they were observed in the roost, from the first capture to the last capture as an adult male.

## Availability of supporting data

The data sets supporting the results of this article are included within the article and its additional files.

## Additional files

10.1186/s12898-016-0056-1: Data set of total group size (adults and juveniles) for 81 capture events at 14 social groups. The columns respectively represent the capture site (1), the capture date (2), the capture month (3), and the total group size (adults and juveniles) (4).
10.1186/s12898-016-0056-1: Survival dataset with estimated lifespan from 114 recaptures of 70 females analysed with Cox PH models. The columns respectively represent the individual transponder (1), the capture site (2), the capture date (3), the minimum survival time in days (4), the estimated survival time from birth in days (5), the censoring of the observation (6), the total group size at previous capture (adults and juveniles) (7), and the adult group size at previous capture (8).
10.1186/s12898-016-0056-1: Survival matrix of 63 adult females analysed with MSMR models. The columns represent respectively the 15 months of recording with absence or group size (1-15), the sex (16), the age (17), the year of first marking (18), and the capture site (19).
